# Core-Shell and Hollow Particles of Carbon and SiC Prepared from Hydrochar

**DOI:** 10.3390/ma12111835

**Published:** 2019-06-06

**Authors:** Wenming Hao, Yongsheng Liu, Alexandra Neagu, Zoltan Bacsik, Cheuk-Wai Tai, Zhijian Shen, Niklas Hedin

**Affiliations:** 1 College of Chemistry and Chemical Engineering, Taiyuan University of Technology, Taiyuan 030024, Shanxi, China; haowenming@tyut.edu.cn; 2 Department of Materials and Environmental Chemistry, Arrhenius Laboratory, Stockholm University, SE-106 91 Stockholm, Sweden; yongshengliu@nwpu.edu.cn (Y.L.); alm.neagu@gmail.com (A.N.); zoltanb@mmk.su.se (Z.B.); cheuk-wai.tai@mmk.su.se (C.-W.T.)

**Keywords:** hollow spheres, silicon carbide, hydrothermal carbonization, pulse current treatment, silicon infiltration

## Abstract

The applications of silicon carbide (SiC) include lightweight materials with thermal shock resistance. In this study, core-shell C-SiC particles were synthesized by compacting and rapidly heating a hydrochar from glucose by using strong pulsed currents and infiltration of silicon vapor. Hollow particles of SiC formed on removing the carbon template. In contrast to related studies, we detected not only the pure 3C polytype (β-SiC) but also significant amounts of the 2H or the 6H polytypes (α-SiC) in the SiC.

## 1. Introduction

Hydrothermal carbonization is an old approach to treat biomass [1,2] that has gotten new attention. In that process, the components of biomass, especially sugars [3] and polysaccharides, are dehydrated into hydrochars [4,5]. The process has a good carbon economy [6], tunable chemistry, and the hydrochars are non-aromatic when the carbonization temperature is relatively low. The hydrochars have been refined into activated carbons [7,8,9,10], supercapacitor electrodes [11], pyrolyzed carbons [12], used to prepare hollow particles of metal oxides at low temperatures [13], and studied for energy applications [14].

Not only are hollow metal oxides interesting for applications, but also hollow SiC is a relevant lightweight material [15], which could provide thermal-shock resistance [16]. Hollow particles of SiC have been synthesized by different protocols [17,18,19,20,21,22,23,24], which include the use of carbon templates and vapor infiltration of silicon or SiO [18,19,20,21,22]. Carbon black is commonly used as carbon template, which reacts with vaporized silicon or SiO, forming SiC on the shell. Hollow SiC particle is obtained when the carbon core is oxidatively removed [18,21]. Silicon carbide (SiC) is mass-produced and has a high temperature stability [25,26,27,28,29,30,31]. With its outstanding thermal, optical, and electrical properties, SiC is a good candidate material for preparing high-frequency transistors, high-temperature sensors, and high-power devices [32,33]. Nanocrystalline powders could, in general, allow for doping and stabilization of unusual structures [34], and rapid sintering of small SiC particles seems to reduce grain-boundary migration. [35] Motivated by research showing that hydrochars from sugars are often observed in the form of spherical and carbon-rich particles [36], and that hollow particles of SiC have been shown to be possible to synthesize from carbon-rich spherical particles, we here used a sustainable hydrochar precursor to synthesize hollow particles of SiC.

## 2. Experimental Section 

### 2.1. Precursors

Glucose (CAS no. 50-99-7; 99.5% purity, Sigma, Saint Louis, MO, USA) and silicon powder (CAS no. 7440-21-3; 99% purity, 325 mesh, Aldrich, Saint Louis, MO, USA) were used without any further purification.

### 2.2. Experimental Procedure

#### 2.2.1. Hydrothermal Carbonization 

Sixty grams of glucose was dissolved in 100 mL of distilled water and then transferred to a 200 mL autoclave. The solution was heated to 200 °C under autogenous pressure for 24 h. The black hydrochar powder was filtered off and washed five times with distilled water, before it was dried at 100 °C overnight. 

#### 2.2.2. Pulse Current Treatment 

The hydrochar powder was consolidated into a disc using a Dr Sinter 2050 spark plasma sintering facility (Sumitomo Coal Mining Co., Ltd., Tokyo, Japan). The powder was loaded into a graphite die with an inner diameter of 12 mm and then subjected to a uniaxial pressure of 50 MPa. Strong pulsed currents (up to 1000 A) heated up the entire setup rapidly. The temperature of the assembly was increased to 100 °C from room temperature with a rate of 50 °C/min and held there for three minutes and then cooled down. 

#### 2.2.3. Infiltration of Vaporized Silicon 

Four grams of a silicon powder was put above and under the hydrochar (0.5 g) disc and separated by a graphite felt in a graphite crucible. It was heated by pulsed currents from room temperature to 600 °C within three minutes under dynamic vacuum at a pressure <10 Pa. Thereafter, the temperature was rapidly increased to 1400 °C with a rate of 200 °C/min and held for five minutes. Subsequently, the temperature was increased to 1600 °C with a rate of 50 °C/min and held there for five minutes. After this treatment, the system was cooled down by natural convection.

#### 2.2.4. Fabrication of Hollow Particles of SiC by Template Removal 

Hollow particles of SiC were fabricated by removing the unreacted carbon from the silicon-infiltrated hydrochar in a muffle furnace at 700 °C in a flow of air for 16 h. 

### 2.3. Characterization 

#### 2.3.1. Elemental Analyses 

Carbon, hydrogen, and nitrogen elements (CHN) analyses were conducted by using combustion analysis. The Si content was analyzed by a Varian Vista MPX (Palo Alto, CA, USA) inductively coupled plasma optical emission spectrometry system, and the oxygen content was calculated as the difference. 

#### 2.3.2. Thermogravimetric Analyses (TGA) 

The mass losses of the hydrochar and core-shell C-SiC composites were studied with a Perkin Elmer TAG7 instrument (Waltham, MA, USA) at 20–900 °C in dry air with a heating rate of 10 °C/min. A platinum cup was used.

#### 2.3.3. N_2_ Adsorption and Textual Analyses 

Analyses were performed at −196 °C with a Micromeritics ASAP 2020 device (Norcross, GA, USA). Samples were degassed at 300 °C for five hours. Specific surface areas were determined from the N_2_ adsorption data at relative pressures of 0.05–0.25 using the Brunauer, Emmett and Teller (BET) model.

#### 2.3.4. Argon Ion-Beam Cross-Section Polishing 

Argon ion-beam cross-section polishing was performed on the core-shell C-SiC composite using an SM-09010 Cross-Section Polisher (JEOL, Tokyo, Japan) with an accelerating voltage of 5 kV with beam currents of 70–90 μA for 15 h. The disc of the core-shell C-SiC composite was fixed on the sample holder of the cross-section polishing apparatus using a carbon paint (Conductive Carbon Cement, Plano, Wetzlar, Germany) during the polishing. 

#### 2.3.5. Scanning Electron Microscopy 

A JEOL JSM-7000F microscope was used to record scanning electron microscopy (SEM in the SE mode) images with a working distance of 10 mm and an accelerating voltage of 5 kV. The sample discs were crushed, and cross sections were imaged by fixing the discs onto Oxford aluminum stubs that were coated subsequently by a thin layer of dried colloidal carbon.

#### 2.3.6. Transmission Electron Microscopy 

The transmission electron microscopy (TEM) experiments were performed at room temperature using a JEOL JEM-2100F microscope (Cs = 0.5 mm) with a Schottky-type field emission gun operated at 200 kV. Samples for TEM observations were crushed and dispersed in absolute ethanol, followed by ultrasonification for 1 min. A droplet of the suspension was transferred onto a copper grid coated with holey carbon film and dried in air.

#### 2.3.7. Infrared Spectroscopy 

Infrared (IR) spectra were collected on a Varian 670-IR IR spectrometer using a Golden Gate attenuated total reflectance (ATR) accessory and a room-temperature detector. Spectra (400–4000 cm^−1^) were recorded with a resolution of 4 cm^−1^.

#### 2.3.8. Raman Spectroscopy 

Raman spectra were recorded on a LabRAM HR 800 Raman spectrometer (Paris, France) with a resolution of 0.5 cm^−1^ using an air-cooled double-frequency Nd:YAG laser (50 mW, 532 nm). The frequency was calibrated to the band frequency of 520.7 cm^−1^ of a silicon wafer. 

#### 2.3.9. X-ray Diffraction 

X-ray diffraction (XRD) data were collected using an X’PERT-PRO PANalyical powder diffractometer (Phillips Company, Amsterdam, The Netherlands) with an X’Celerator detector (CuKa1 radiation, k = 1.5418 Å) between 2θ = 20.0–80.0° in its reflection mode.

## 3. Results and Discussion

**Figure 1:**
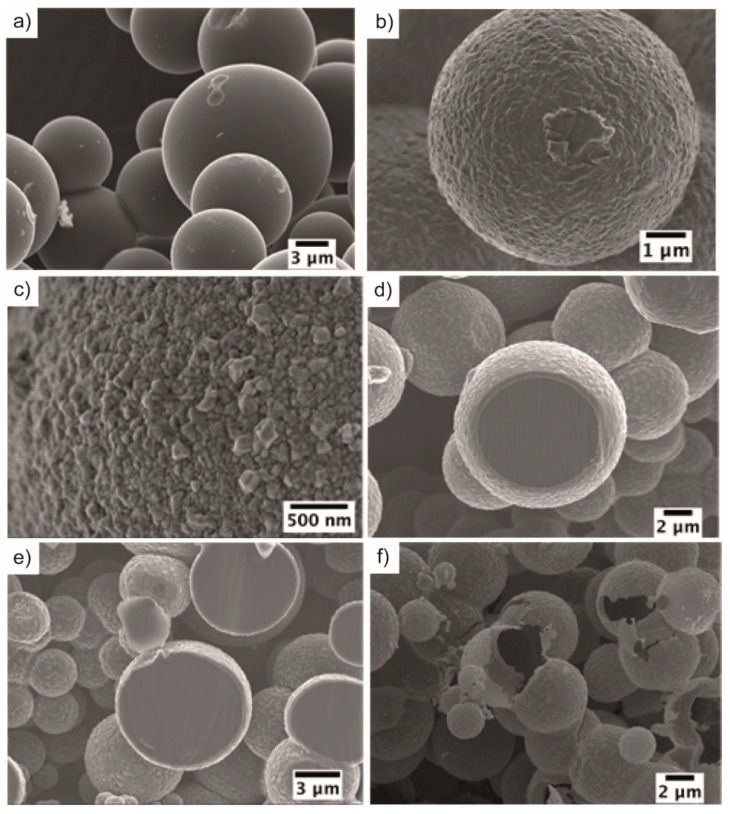
SEM images of (**a**) hydrochar of glucose, (**b**) core-shell C-SiC particles, (**c**) the surface of a core-shell particle, (**d**,**e**) cross-sections of core-shell particles, (**f**) hollow particles of SiC.

A hydrochar disc was prepared from a hydrochar powder, using a protocol developed by us [37], which was subsequently used as a precursor for the synthesis of core-shell particles of C-SiC and hollow particles of SiC. The hydrochar powder had a typical composition with significant amounts of C and O (see Table 1) [38]. Such hydrochars consist mainly of solid furan-rich polymers [39] and an acetone-soluble liquid fraction [37,40]. The diameters of the hydrochar particles were 5–10 µm (see Figure 1a), and the comparably polydispersed nature related to the comparably long reaction time (24 h) and high concentration of gluc4ose used for the synthesis. 

**Figure 2:**
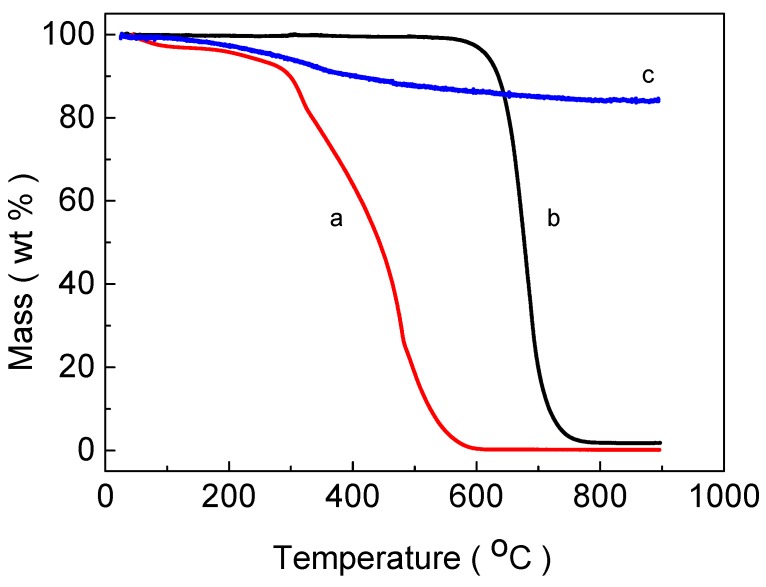
Thermogravimetric curves of hydrochar of glucose (**a**), core-shell C-SiC particles (**b**), hollow SiC particles (**c**) recorded in dry air.

The hydrochar powder was not very porous with a BET surface area of 8 m^2^/g. The water content was ~4 wt % as estimated from the mass loss observed at a temperature of 110 °C, as presented in Figure 2a. A dramatic mass loss was observed at temperatures >200 °C and related to evaporation of volatiles and decomposition of the condensed structure of the hydrochar [41,42].

The hydrochar disc was carbonized and used as both the sacrificial template and source of carbon for core-shell C-SiC particles subsequently transformed into hollow particles of SiC. Compared with other carbon sources, hydrochar can be prepared from biomass as a renewable source via a sustainable approach. Silicon vapor was infiltrated into the hydrochar discs, and a shell of SiC formed on the hydrochar. The hydrochar and the core-shell C-SiC particles had similar morphologies, as can be observed from Figure 1a,b. Similar and related transfer of morphological features have been observed for pyrolyzed lignocellulosic structures transformed into C-SiC particles on silicon vapor infiltration [43]. 

**Figure 3:**
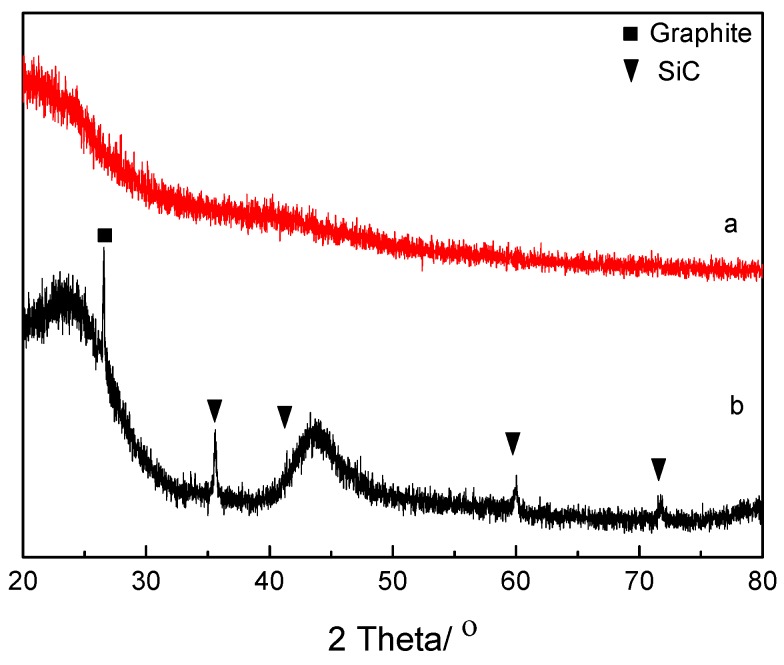
X-ray diffractograms of hydrochar from glucose (**a**) and a core-shell C-SiC composite (**b**). The broad peaks at about 24° and 44° are typical for amorphous carbon.

The core-shell C-SiC particles had small crystals (20–200 nm) of SiC on their surfaces assembled into thin shells with thicknesses of 300–500 nm, as shown in Figure 1c–e. The SiC particles seemed to consist of β-SiC from the XRD pattern in Figure 3b. The XRD pattern had narrow and broad peaks of amorphous carbon, some graphite, and narrow peaks from β-SiC. The broad X-ray peaks at 24° and 44° were typical for partially graphitized amorphous carbon [44,45]. The hydrochar powder was, on the other hand, X-ray amorphous (Figure 3a). 

The core-shell particles of C-SiC contained 3 wt % of SiC (Table 1) and had a BET-specific surface area of 10 m^2^/g. The TGA trace was typical of a comparably stable structure, as can be seen from the trace in Figure 2b. No mass loss was observed at temperatures <600 °C, which indicated that the carbon core was stable at these temperatures. The sharp decrease in weight observed at temperatures of 650–700 °C was attributed to an oxidative removal of the carbon core. The weight of the sample stabilized at temperatures >800 °C with a residue attributed to SiC. 

**Figure 4:**
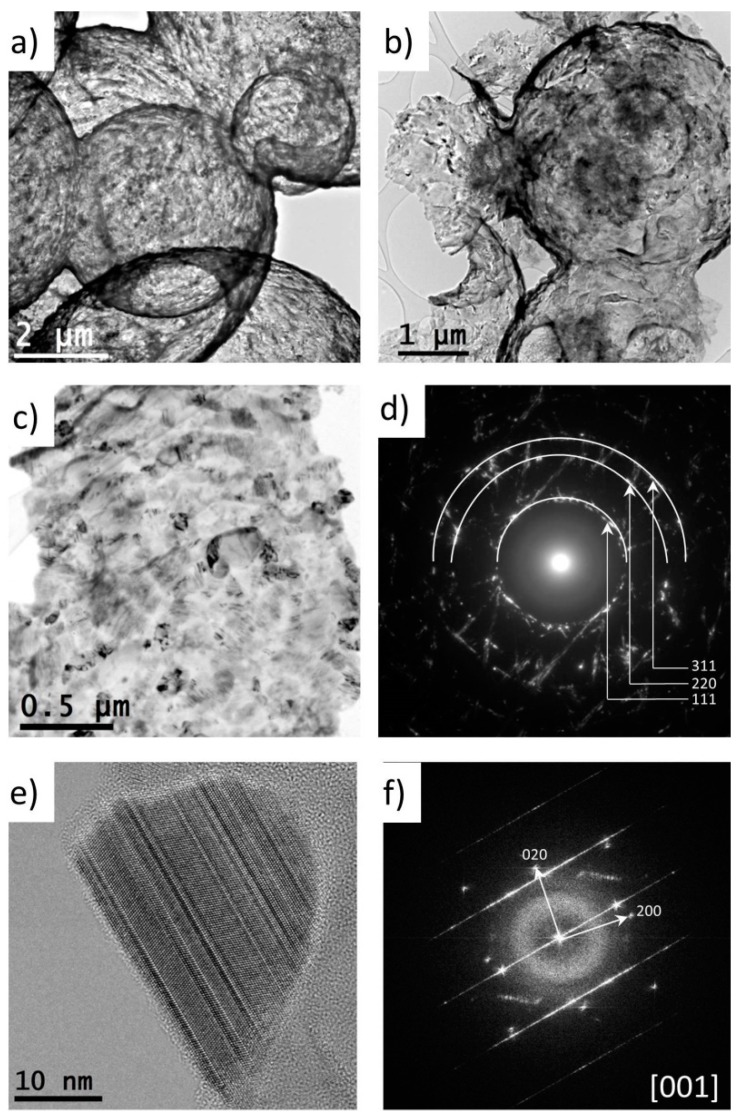
TEM images of hollow spheres of SiC: (**a**) and (**b**) Bright-field TEM image showing an overview of the hollow spheres’ morphology; (**c**) TEM image of polycrystalline grains from which the hollow shell is formed; (**d**) the corresponding selected-area electron diffraction (SAED) pattern; (**e**) HRTEM image of a single grain showing a high density of stacking faults; (**f**) the corresponding fast Fourier transform.

The carbon cores of the core-shell particles were removed at a temperature of 700 °C in a flow of air using a protocol similar to those of Ye et al. and Zhang et al. [18,19]. A longer oxidation time was used as compared with the study of Zhang et al. [19], as the underlying particles were larger in our case. After oxidation, the sample contained hollow and interconnected particles of SiC, as can be seen in Figure 1f (SEM) and Figure 4a (TEM). The TG curve in Figure 2c showed that the hollow SiC particle had 16 wt % of weight loss at 900 °C, which could probably be attributed to adsorbed moisture and some remaining carbon content.

Representative low-magnification TEM images illustrating the general morphology of the hollow particles of SiC are shown in Figure 4a,b. The strong contrast between the dark edge and the light area in the center further confirmed the hollow nature after the oxidation of the carbon core. We observed a relatively broad size distribution of the hollow spheres with the diameter ranging from 2.5–6 µm, which was consistent with the particle distribution of the hydrochar template. Small amounts of impurities were found, indicative of traces of amorphous SiO_2_. The polycrystalline nature of the SiC in the shells was studied by selected-area electron diffraction (SAED). Typical TEM images of the polycrystalline SiC grains in the shells and the corresponding SAED pattern are shown in Figure 4c,d. The grains had a broad size distribution, and the SAED pattern indicated a high density of defects, such as twinning and stacking faults, as indicated by streaking. The SAED pattern had diffraction rings characteristic of a polycrystalline phase indexed as the cubic 3C-SiC structure (F-43m), as is illustrated in Figure 4d. However, because of the high density of defects, the presence of other polytypes of SiC (like 6H-SiC) could not be excluded [46]. High-resolution TEM (HRTEM) images were recorded to further investigate the crystallinity and the type of defects in the grains. The shells consisted of highly-defective crystalline grains, as can be observed in a typical HRTEM image (Figure 4e) and a corresponding fast Fourier transform (Figure 4f). A large density of stacking faults was observed. Most of the grains were defective, and the TEM study could not give a full answer about the corresponding polymorphism. 

**Figure 5:**
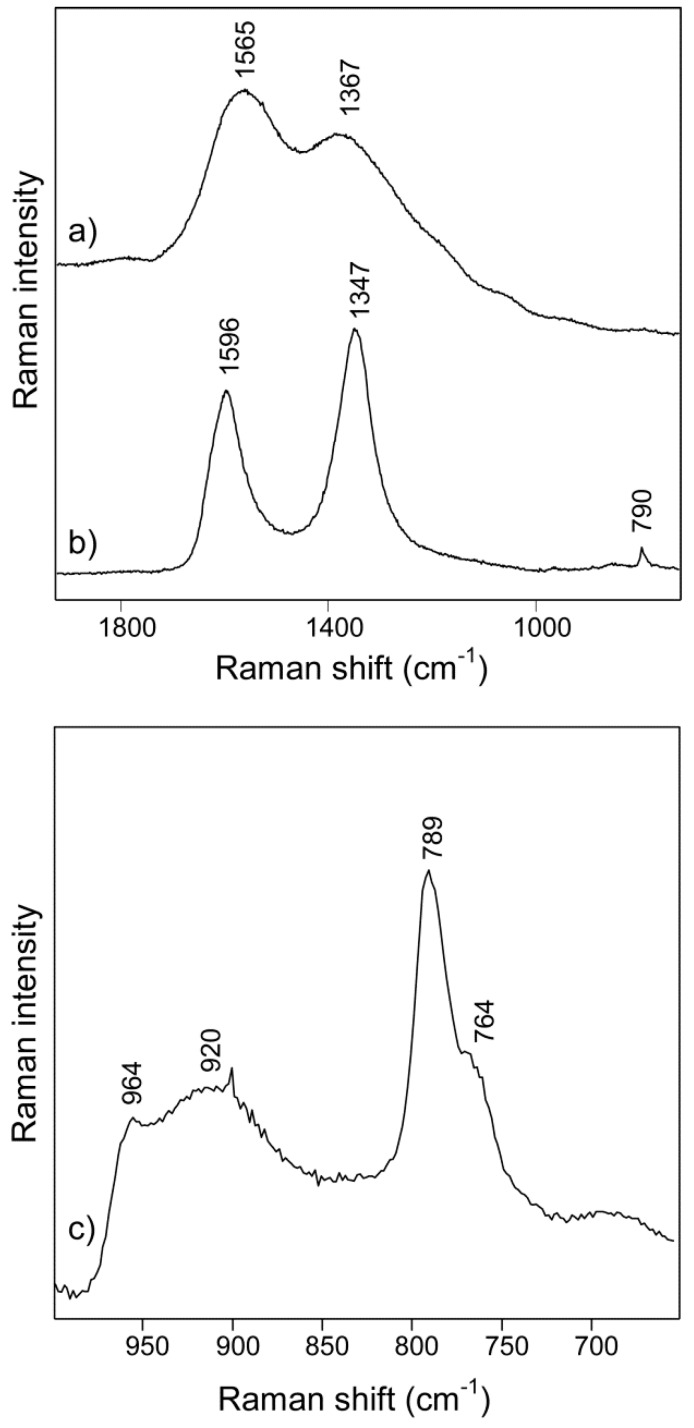
Raman spectra of (**a**) hydrochar of glucose, (**b**) the core-shell C-SiC composite and (**c**) the hollow and spherical SiC particles.

The Raman spectrum of the hydrochar (in Figure 5a) is very similar to those in literature [5,47]. The band at 1367 cm^−1^ was assigned to either the ring-breathing vibration in aromatic structures or vibrations relating to sp^3^-hybridized carbons. The band at about 1590 cm^−1^ (the G-band) was assigned to the C-C stretching vibrations of sp^2^-hybridized carbon atoms. However, it was not judged relevant to characterize the hydrochars with the G/D-ratio. The band in the D-band regime of the hydrochars was not the defect-derived band of aromatics, as would be typically observed for amorphous carbons or graphite prepared at much higher temperatures than the hydrochars. The Raman spectrum of the core-shell C-SiC composite particles in Figure 5b, on the other hand, had the typical D (with a band at about 1347 cm^−1^) and G-bands (1596 cm^−1^), with ratios being typical for amorphous carbon materials [48], and a band at 790 cm^−1^ from the Si-C stretching. The Raman spectrum in Figure 5c of the hollow particles of SiC was typical for SiC [49,50]. The bands were broad and typical for disordered crystals [49]. To determine the polytype of the SiC, investigating the bands at low wavenumbers (so-called FTA modes) would have been most useful. However, only a weak band at 262 cm^−1^ was detected (not shown in the figure). Considering the position of the bands detected (FTO modes) at 964, 789, 764, and 262 cm^−1^, an attempt was made to determine the polytype of the SiC. According to these band positions, a pure 3C polytype (β-SiC) nature was clearly excluded [49]. The spectrum was most similar to the 2H polytype. Other polytypes, especially the 6H polytype with similar band intensities at around 790/760 cm^−1^ and with the most intense FTA mode at around 266 cm^−1^, could not be fully excluded because of the broad bands. The spectra were reproducible when being recorded at different spots of the sample. The very broad underlying band with maximum around 920 cm^−1^ was likely related to the impurity component of SiO_2_. However, assignments to SiC polytypes were not fully excluded as the frequency was slightly lower than commonly observed for SiO_2_. In contrast to other studies of hollow particles of SiC [17,18,20,22,23,24], we did not detect the pure 3C polytype (β-SiC). Instead, the spectra were consistent with highly defective and polycrystalline SiC of the 2H or possibly the 6H (α-SiC) polytype. Raman spectroscopy is, in our view, needed to be used when investigating the polymorphisms in SiC samples, such as in this study. Note that Liu et al. have shown that β-SiC can form together with an amorphous layer [21]. SiC with different polytypes can give different properties. It was reported that β-SiC and α-SiC have different behavior at high temperature [30,31]. 

**Figure 6:**
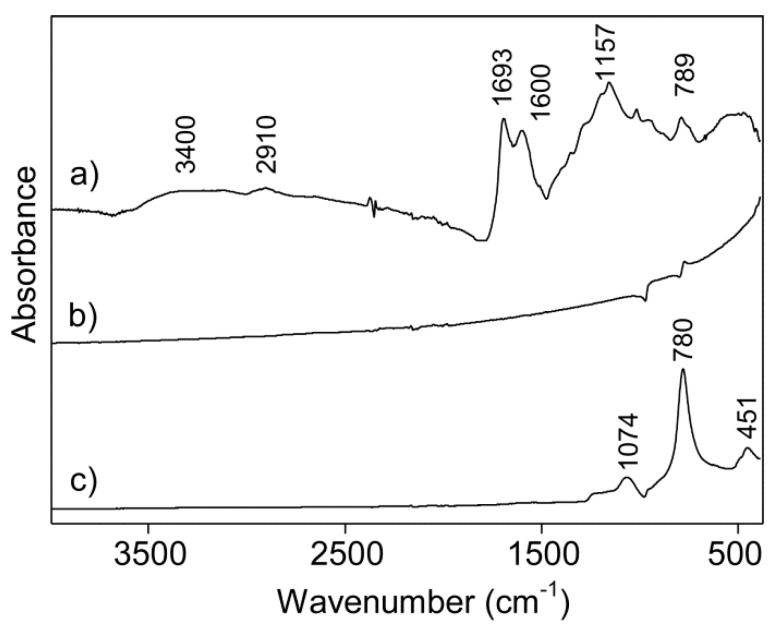
IR spectra of (**a**) hydrochar prepared from glucose, (**b**) a core-shell C-SiC composite, and (**c**) hollow and spherical SiC particles.

The functional groups of the hydrochar, core-shell C-SiC, and hollow particles of SiC were studied by IR spectroscopy. The IR spectrum of the hydrochar is presented in Figure 6a. The broad band at 3600–3000 cm^−1^ were attributed to the O-H stretching vibrations of water, alcohols, or phenols groups; the band at 2910 cm^−1^ was assigned to the C-H stretching vibrations of aliphatic alkyl chains [51]; and the bands centered at 1693 and 1600 cm^−1^ were associated to the C=O stretching vibration of the carbonyl and carboxylic groups. The latter one could also be assigned to C=C stretching [52]. The bands in the 1300–1000 cm^−1^ region were assigned to mainly C-O stretching vibrations in alcohols, phenols and carboxyl groups, and the band centered at 790 cm^−1^ was related to the out-of-plane bending vibration of aromatic C-H groups [53] and was indicative of some aromatization of the hydrochar. The IR spectrum of the core-shell C-SiC composite basically lacked band features (Figure 6b) after the high-temperature treatment and vapor infiltration of silicon. Pure carbons do not have bands in the IR spectra, and the shape of the baseline in Figure 6b is typical for scattering of IR radiation by carbons. The two derivative-like spectral features of this spectrum likely related to bands of SiC. The derivative-like shape of these bands was a consequence of the various interactions between the IR radiation and the core-shell C-SiC material. This assignation of the derivative-like features was consistent with the features of the IR spectrum of the hollow SiC spheres in Figure 6c. The spectrum in Figure 6c agreed well with those previously being reported for SiC [54]. The IR band shapes and, to a certain extent, band positions have been shown to depend on the size of the particles but cannot provide information about the polytype of the SiC [54]. The band at 780 cm^−1^ corresponded to the Si-C stretching vibrations, and the bands at 1074 and 451 cm^−1^ were firmly assigned to the Si-O stretching and bending vibrations for the SiO_2_ impurities also being observed by features in certain TEM images.

## 4. Conclusions

Hollow and spherical particles of SiC were prepared using a precursor powder of hydrothermally carbonized glucose. The hydrochar was subjected to infiltration of silicon vapor and a subsequent oxidative removal of unreacted carbon. The spherical morphology of the hydrochar particles was transferred to the spherical shells of SiC. Analyses of the TEM and X-ray diffraction data indicated that the SiC was of the 3C polytype (β-SiC), but the features in the Raman spectrum distinctively excluded a pure β-SiC composition of the defective nanocrystals in the thin SiC shells. The Raman spectrum was consistent with significant fractions of the 2H or 6H polytypes of SiC (α-SiC).

In future work, the thickness of the SiC shell could be controlled by the time of silicon vapor infiltration or by using chemical vapor infiltration. The size of the hollow SiC particles could likely be controlled by varying the size of the hydrochar particles. In addition to such variations, it could be relevant to also test the mechanical and thermal properties of macroscopic samples of the intergrown and hollow particles of SiC. 

## Figures and Tables

**Table 1:** Brunauer, Emmett and Teller (BET) surface area, bulk element composition of the hydrochar prepared from glucose (HTC), and the core-shell C-SiC composite.

Sample	S_BET_ (m^2^/g)	Ultimate (wt %)					
		C	H	N	O ^a^	Si	
HTC	8	66.2	4.3	<0.10	29.5	-	-
Core-shell-C-SiC	10	98.2	<0.10	<0.10	1.47	0.13 ^b^	1.40 ^c^

^a^ calculated by difference; ^b^ estimated by elemental analysis; ^c^ estimated by TGA.
